# Geobiology of Andean Microbial Ecosystems Discovered in Salar de Atacama, Chile

**DOI:** 10.3389/fmicb.2021.762076

**Published:** 2021-10-28

**Authors:** Federico A. Vignale, Daniel Kurth, Agustina I. Lencina, Daniel G. Poiré, Elizabeth Chihuailaf, Natalia C. Muñoz-Herrera, Fernando Novoa, Manuel Contreras, Adrián G. Turjanski, María E. Farías

**Affiliations:** ^1^Laboratorio de Investigaciones Microbiológicas de Lagunas Andinas (LIMLA), Planta Piloto de Procesos Industriales Microbiológicos (PROIMI), CCT, CONICET, San Miguel de Tucumán, Argentina; ^2^Laboratorio de Bioinformática Estructural, Instituto de Química Biológica de la Facultad de Ciencias Exactas y Naturales (IQUIBICEN)-CONICET, Universidad de Buenos Aires, Buenos Aires, Argentina; ^3^Centro de Investigaciones Geológicas (CIG), Universidad Nacional de La Plata (UNLP)-CONICET, La Plata, Argentina; ^4^Centro de Ecología Aplicada (CEA), Santiago, Chile

**Keywords:** Andean lakes, microbial mats, microbialites, endoevaporites, extremophiles

## Abstract

The Salar de Atacama in the Chilean Central Andes harbors unique microbial ecosystems due to extreme environmental conditions, such as high altitude, low oxygen pressure, high solar radiation, and high salinity. Combining X-ray diffraction analyses, scanning electron microscopy and molecular diversity studies, we have characterized twenty previously unexplored Andean microbial ecosystems in eight different lakes and wetlands from the middle-east and south-east regions of this salt flat. The mats and microbialites studied are mainly formed by calcium carbonate (aragonite and calcite) and halite, whereas the endoevaporites are composed predominantly of gypsum and halite. The carbonate-rich mats and microbialites are dominated by *Bacteroidetes* and *Proteobacteria* phyla. Within the phylum *Proteobacteria*, the most abundant classes are *Alphaproteobacteria*, *Gammaproteobacteria* and *Deltaproteobacteria*. While in the phylum *Bacteroidetes*, the most abundant classes are *Bacteroidia* and *Rhodothermia*. *Cyanobacteria*, *Chloroflexi*, *Planctomycetes*, and *Verrucomicrobia* phyla are also well-represented in the majority of these systems. Gypsum endoevaporites, on the contrary, are dominated by *Proteobacteria*, *Bacteroidetes*, and *Euryarchaeota* phyla. The *Cyanobacteria* phylum is also abundant in these systems, but it is less represented in comparison to mats and microbialites. Regarding the eukaryotic taxa, diatoms are key structural components in most of the microbial ecosystems studied. The genera of diatoms identified were *Achnanthes*, *Fallacia*, *Halamphora*, *Mastogloia*, *Navicula*, *Nitzschia*, and *Surirella*. Normally, in the mats and microbialites, diatoms form nano-globular carbonate aggregates with filamentous cyanobacteria and other prokaryotic cells, suggesting their participation in the mineral precipitation process. This work expands our knowledge of the microbial ecosystems inhabiting the extreme environments from the Central Andes region, which is important to ensure their protection and conservation.

## Introduction

**FIGURE 1:**
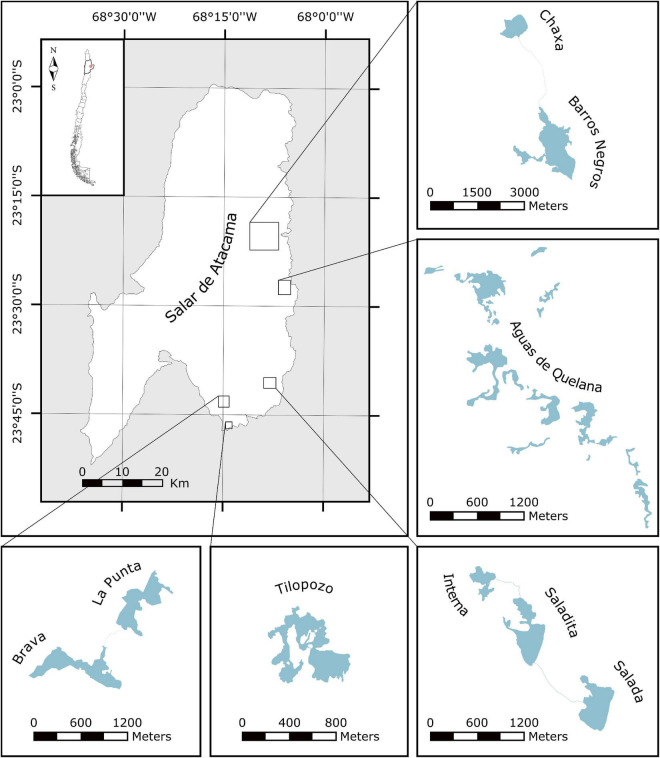
Location of the study area which consists of a system of several lakes and wetlands. The eight lakes and wetlands of this study are outlined in the five respective inserts.

The Salar de Atacama (Figure 1), located in the Chilean Central Andes, is one of the Earth’s largest evaporite basins (ca. 3,000 km^2^) (Alonso and Risacher, 1996). It comprises numerous hypersaline lakes, locally called “lagunas,” at the edge of which evaporitic crusts form. A combination of extreme environmental conditions, such as high altitude, low barometric pressure, high solar and UV radiation, low rates of precipitation, high rates of evaporation, high salinity, strong winds, wide daily range in temperatures and the occurrence of hydrothermal activity makes this area one of the best primitive Earth analogs. Consequently, the study of the Andean microbial ecosystems (AMEs) that thrive in this environment may help us improve our understanding of the earliest complex ecosystems on Earth, as well as provide information for the search for life on other planets (Cabrol et al., 2009; Vignale et al., 2021).

In recent years, a variety of AMEs have been reported and studied in the Salar de Atacama. These include microbial mats at Laguna Cejar (∼2,300 m a.s.l.) (Rasuk et al., 2016), Laguna Tebenquiche (∼2,340 m a.s.l.) (Thiel et al., 2010; Farías et al., 2014; Fernandez et al., 2016; Kurth et al., 2021), Laguna Chaxa (∼2,300 m a.s.l.) (Thiel et al., 2010), and Laguna Brava (∼ 2,300 m a.s.l.) (Farías et al., 2014; Farias et al., 2017; Kurth et al., 2021); microbialites at Laguna Brava (Farías et al., 2014; Farias et al., 2017) and Laguna Interna (∼2,300 m a.s.l.) (Osman et al., 2021); and gypsum endoevaporites at Laguna de la Piedra (∼2,300 m a.s.l.) (Stivaletta et al., 2011) and Laguna Tebenquiche (Farías et al., 2014; Fernandez et al., 2016). Microbial mats are vertically laminated biofilms that occasionally lithify, forming organosedimentary structures called microbialites (Stolz, 2000). The mineralization of mats can be microbially induced or microbially influenced. In the former, microbial metabolic activities (e.g., photosynthesis, sulfate reduction, nitrate-driven sulfide oxidation) induce conditions for mineral precipitation, while in the latter, external environmental processes (e.g., degassing, evaporation) create the conditions for mineral precipitation. An organic matrix comprised of extracellular polymeric substances (EPS) is, however, involved in both microbially mediated mineralizations providing a template for mineral nucleation (Dupraz and Visscher, 2005; Dupraz et al., 2009). In the high-altitude Andean lakes (HAALs) of the Central Andes, both mineralization processes take place (Gomez et al., 2014, 2018; Vignale et al., 2021). Evaporites, on the other hand, are salt deposits precipitated from a saturated brine via solar evaporation (Warren, 2016). Occasionally, they present endolithic microbial communities named endoevaporites. The minerals that form the evaporites, mainly halite (NaCl) and gypsum (CaSO_4_⋅2H_2_O), provide protection against desiccation and UV-B radiation but allow photosynthetically active radiation (400–700 nm) to pass through, which is important for the development of photoautotrophs (Stivaletta et al., 2011; Rasuk et al., 2014; Fernandez et al., 2016; Vignale et al., 2021).

The aim of this study is to expand our knowledge of the AMEs in the extreme environments of Salar de Atacama. To fulfill this objective, first we identified previously unknown microbial ecosystems (biofilms, microbial mats, microbialites, and endoevaporites) in eight different lakes and wetlands from Salar de Atacama, and then we analyzed the composition of their mineral phase and microbiota. Finally, we performed comparative analyses of the data to determine if there was a correlation between these features.

## Materials and Methods

### Sample Collection

In the first place, a delimitation of the study area (Figure 1) was performed through the analysis of satellite and aerial images obtained from Google Earth and a DJI^®^ Mavic Pro drone, respectively. Afterward, three field campaigns (24–28 November 2017, 24–26 November 2018, and 17 May 2018) were carried out to explore the respective area and search for AMEs.

In total, twenty previously undescribed AMEs were identified in eight different lakes and wetlands from the middle-east and south-east regions of Salar de Atacama. These are a floating biofilm from Tilopozo (T); microbial mats from Aguas de Quelana (Q1 and Q2), Laguna Salada (S1), Laguna La Punta (P1 and P2) and Laguna Brava (B1); microbialites from Laguna Chaxa (CH1, CH2, and CH3), Laguna Interna (I), Laguna La Punta (P3) and Laguna Brava (B2, B3, and B4); endoevaporites from Laguna Barros Negros (BN1, BN2, and BN3); a microbial community within an Andean flamingo (*Phoenicoparrus andinus*) mound nest from Laguna Salada (S2) and a sediment-associated microbial community from Aguas de Quelana (Q3).

Bulk samples were taken from the respective AMEs to perform mineralogy and scanning electron microscopy (SEM) analyses. These samples were stored in the dark at 4°C, and processed within 1–2 weeks. Additionally, samples were taken from different layers of the AMEs to perform 16S rRNA diversity analyses (Table 1). These samples were stored in RNAlater^®^ solution (Thermo Fisher Scientific, United States) at 4°C in the dark, and processed within a week.

**TABLE 1:** Overview of samples used to perform 16S rRNA diversity analyses, locations and coordinates.

**Sample label**	**Lake/Wetland**	**Sample description**	**GPS location [UTM]**	**Elevation [m]**
CH1	Chaxa	Biofilm over microbialite	584877 E, 7419856 N	2,305
CH2A	Chaxa	Microbial mat over microbialite, first layer	584754 E, 7419856 N	2,305
CH2B	Chaxa	Microbial mat over microbialite, second layer	584754 E, 7419856 N	2,305
CH2C	Chaxa	Microbial mat over microbialite, third layer	584754 E, 7419856 N	2,305
CH3	Chaxa	Biofilm over microbialite	584877 E, 7419709 N	2,305
BN1A	Barros Negros	Endoevaporite, first layer	584580 E, 7416203 N	2,305
BN1B	Barros Negros	Endoevaporite, second layer	584580 E, 7416203 N	2,305
BN1C	Barros Negros	Endoevaporite, third layer	584580 E, 7416203 N	2,305
BN2A	Barros Negros	Endoevaporite, first layer	586132 E, 7416056 N	2,305
BN2B	Barros Negros	Endoevaporite, second layer	586132 E, 7416056 N	2,305
BN3A	Barros Negros	Endoevaporite, first layer	586132 E, 7416056 N	2,305
BN3B	Barros Negros	Endoevaporite, second layer	586132 E, 7416056 N	2,305
BN3C	Barros Negros	Endoevaporite, third layer	586132 E, 7416056 N	2,305
Q1A	Aguas de Quelana	Microbial mat, first layer	592225 E, 7408159 N	2,305
Q1B	Aguas de Quelana	Microbial mat, second layer	592225 E, 7408159 N	2,305
Q1C	Aguas de Quelana	Microbial mat, third layer	592225 E, 7408159 N	2,305
Q2A	Aguas de Quelana	Microbial mat, first layer	592048 E, 7405582 N	2,305
Q2B	Aguas de Quelana	Microbial mat, second layer	592048 E, 7405582 N	2,305
Q2C	Aguas de Quelana	Microbial mat, third layer	592048 E, 7405582 N	2,305
Q3	Aguas de Quelana	Sediment	592248 E, 7409988 N	2,305
IA	Interna	Microbial mat over microbialite, first layer	586221 E, 7382261 N	2,305
IB	Interna	Microbial mat over microbialite, second layer	586221 E, 7382261 N	2,305
IC	Interna	Microbial mat over microbialite, third layer	586221 E, 7382261 N	2,305
S1A	Salada	Microbial mat, first layer	587747 E, 7380714 N	2,305
S1B	Salada	Microbial mat, second layer	587747 E, 7380714 N	2,305
S2A	Salada	Endolithic microbial community inhabiting an Andean flamingo mound nest, first layer	587635 E, 7380585 N	2,305
S2B	Salada	Endolithic microbial community inhabiting an Andean flamingo mound nest, second layer	587635 E, 7380585 N	2,305
T	Tilopozo	Floating biofilm	577847 E, 7370337 N	2,312
P1A	La Punta	Microbial mat associated with vegetation, first layer	578154 E, 7376594 N	2,305
P1B	La Punta	Microbial mat associated with vegetation, second layer	578154 E, 7376594 N	2,305
P1C	La Punta	Microbial mat associated with vegetation, third layer	578154 E, 7376594 N	2,305
P2A	La Punta	Microbial mat associated with vegetation, first layer	577965 E, 7376601 N	2,305
P2B	La Punta	Microbial mat associated with vegetation, second layer	577965 E, 7376601 N	2,305
P2C	La Punta	Microbial mat associated with vegetation, third layer	577965 E, 7376601 N	2,305
P3	La Punta	Biofilm over microbialite	577784 E, 7376362 N	2,305
B1A	Brava	Microbial mat, first layer	576689 E, 7376362 N	2,305
B1B	Brava	Microbial mat, second layer	576689 E, 7376362 N	2,305
B1C	Brava	Microbial mat, third layer	576689 E, 7376362 N	2,305
B2	Brava	Biofilm over microbialite	576689 E, 7375484 N	2,305
B3A	Brava	Microbial mat over microbialite, first layer	575982 E, 7375291 N	2,305
B3B	Brava	Microbial mat over microbialite, second layer	575982 E, 7375291 N	2,305
B4A	Brava	Microbial mat over microbialite, first layer	576459 E, 7375524 N	2,305
B4B	Brava	Microbial mat over microbialite, second layer	576459 E, 7375524 N	2,305
B4C	Brava	Microbial mat over microbialite, third layer	576459 E, 7375524 N	2,305
				

### Bulk Mineralogy Analyses

The mineral composition of the samples was determined by X-ray diffraction (XRD) analyses, which were carried out on finely ground sample material (<20 μm) employing a PANalytical X’Pert Pro Multipurpose Powder Diffractometer, with a Cu X-ray source (*k*α = 1.5403 Å), operated at 40 kV and 40 mA at Centro de Investigaciones Geológicas (CIG), La Plata, Argentina. The dried and ground samples were scanned from 2 to 40 2θ, with a scanning speed of 0.04°/s and a time per step of 0.50 s. For each XRD pattern, semi-quantification of the minerals was obtained from the intensity of the main peaks (Schultz, 1964; Moore and Reynolds, 1989). The mineral composition of the samples was used to perform a chloride, sulfate and carbonate ternary diagram with the R package ggtern (Hamilton and Ferry, 2018).

### Scanning Electron Microscopy (SEM) Analyses

Samples were fixed overnight at 4°C in modified Karnovsky fixative, composed of formaldehyde (8% v/v), glutaraldehyde (16% v/v) and phosphate-buffered saline (PBS; 0.2 M, pH 7.4). Afterward, the samples were washed three times with PBS and calcium chloride (CaCl_2_) for 10 min, and fixed with osmium tetroxide (2% v/v) overnight. Finally, after washing twice with ethanol (30% v/v) for 10 min, the samples were dried at critical point and sputtered with gold. Specimens were observed under vacuum using a Zeiss Supra 55VP (Carl Zeiss NTS GmbH, Germany) scanning electron microscope at Centro Integral de Microscopía Electrónica (CIME), Tucumán, Argentina. Biofilm from Tilopozo could not be imaged using SEM due to difficulties encountered during sample preparation. Identification of diatom taxa, based on morphological features of their frustules, was carried out employing SEM images and identification keys (Cristóbal et al., 2020). The morphological features analyzed were: size, shape and symmetry of valves; presence, location and structure of the axial area; presence, location and structure of raphe; presence of septa; presence and structure of costae; location, structure and density of striae; and presence of stauros, among others.

### DNA Extraction and Amplicon Sequencing

Total genomic DNA was isolated from the samples, detailed in Table 1, using the FastDNA^®^ SPIN Kit for Soil (MP Biomedicals, United States) according to the protocol provided by the manufacturer. DNA yield (ng μl^–1^) was quantified spectrophotometrically with a NanoDrop 2000c Spectrophotometer (Thermo Fisher Scientific, United States). NanoDrop was also used to estimate the purity of the extracted DNA. Sample BN3A could not be further processed as not enough DNA was extracted from it. The hypervariable regions V3 and V4 of the bacterial and archaeal 16S rRNA gene were amplified using the primers Bakt_341F (CCTACGGGNGGCWGCAG) and Bakt_805R (GACTACHVGGGTATCTAATCC). The amplicons obtained were sequenced by Macrogen Inc., (Seoul, South Korea) using an Illumina MiSeq sequencer platform. Raw sequences were submitted to the ENA Project database under the accession number PRJEB44218.

### Bioinformatics

Raw Sequence data were analyzed with the software package QIIME 2 v.2018.2, following the tutorials provided by the developers (Caporaso et al., 2010; Bolyen et al., 2018). Taxonomic annotation was performed in QIIME 2 using the SILVA database (No. 132, April 2018) (Quast et al., 2013) trained with 16S rRNA genes grouped in OTUs with 97% similarity. OTUs classified as coming from chloroplast or mitochondria were removed before further analyses. The R package phyloseq (McMurdie and Holmes, 2013) was used to calculate alpha diversity metrics (Chao1, ACE, Shannon and Simpson) after rarefying the data, and perform a principal coordinate analysis (PCoA) based on Bray-Curtis dissimilarity. The R package vegan (Oksanen et al., 2013) was used to perform a redundancy analysis (RDA) and correlate mineralogy with the microbial composition of the samples. Permutational ANOVA (PERMANOVA) tests were also carried out with the R package vegan to evaluate the PCoA and RDA analyses.

## Results

### Microbial Ecosystems Discovered in Salar de Atacama

The exploration carried out in the middle-east and south-east regions of Salar de Atacama allowed us to identify and study twenty previously unreported AMEs in eight different lakes and wetlands: Laguna Chaxa, Laguna Barros Negros, Aguas de Quelana, Laguna Interna, Laguna Salada, Laguna La Punta, Laguna Brava, and Tilopozo.

**FIGURE 2:**
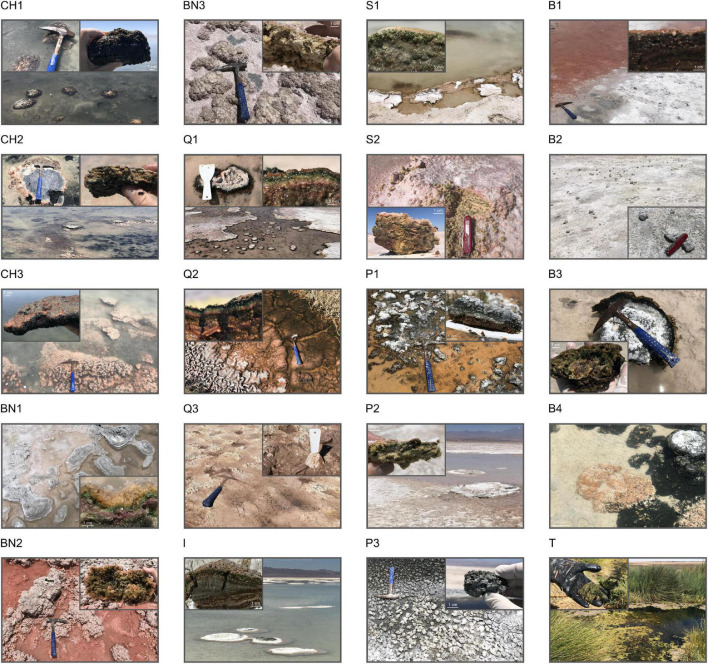
General views of the AMEs discovered in Salar de Atacama. CH1, CH2 and CH3, Microbialites from Laguna Chaxa; BN1, BN2 and BN3, Endoevaporites from Laguna Barros Negros; Q1 and Q2, Microbial mats from Aguas de Quelana; Q3, Sediment-associated microbial community from Aguas de Quelana; I, Microbialite from Laguna Interna; S1, Microbial mat from Laguna Salada; S2, Microbial community within an Andean flamingo mound nest from Laguna Salada; P1 and P2, Microbial mats from Laguna La Punta; P3, Microbialite from Laguna La Punta; B1, Microbial mat from Laguna Brava; B2, B3 and B4, Microbialites from Laguna Brava; T, Floating biofilm from Tilopozo.

Laguna Chaxa is a small lake (∼0.35 km^2^ area) from the middle-east region of Salar de Atacama (Figure 1). A previous study reported the presence of red–purple-colored microbial mats developing in this lake (Thiel et al., 2010). In addition to these microbial mats, we have discovered modern microbialites developing in this environment (Figure 2). Some of these microbialites are covered by black or pink biofilms (CH1 and CH3, respectively), while others are covered by microbial mats that usually display a sequence of (from top to bottom) pink/orange, green and purple colored layers (CH2).

Two kilometers south from Laguna Chaxa is Laguna Barros Negros (∼1.38 km^2^ area) (Figure 1). In this lake, contrary to Laguna Chaxa, we have identified numerous microbial communities developing inside evaporite deposits (endoevaporites) forming colored strata (Figure 2). Normally, a yellow/orange layer (ca. 0.5–2 cm thick) is visible on the top, followed by a green layer of similar thickness (BN2). In some cases, a third purple layer is visible at the bottom as well (BN1 and BN3).

Aguas de Quelana is a wetland complex (∼1.5 km^2^ area) also located in the middle-east region of Salar de Atacama (Figure 1). Neither microbialites nor endoevaporites could be identified in the explored area of this environment. Nevertheless, microbial mats (Q1 and Q2) and pink/orange sediment-associated microbial communities (Q3) were observed along the shorelines (Figure 2). Like the endoevaporites from Laguna Barros Negros, the microbial mats form colored strata. Normally, the first layer (rich in EPS) varies in color and thickness (ca. 0.1–0.3 cm) from one mat to another, while the second and third layers are green and purple, respectively, and usually have a similar thickness (ca. 0.2 cm). Occasionally, a dark layer can be found at the bottom as well. When mats are exposed to the air, a white evaporitic crust covers their surface. Cerebroid, snake and globular morphologies have been observed in these microbial mats, which usually accumulate gas at the subsurface.

Laguna Interna (∼0.08 km^2^ area) and Laguna Salada (∼0.18 km^2^ area) are two connected lakes located in the south-east region of Salar de Atacama (Figure 1). In Laguna Interna, we have discovered modern microbialites (I) that exhibit a laminated mesostructure (stromatolites), covered by microbial mats that usually display a sequence of (from top to bottom) pink/orange, green and purple colored layers (Figure 2). These microbialites are similar to those recently reported in this environment (Osman et al., 2021). In Laguna Salada, on the contrary, we have identified microbial mats (S1) that form colored strata (Figure 2). Typically, a pink/orange layer (ca. 0.1–0.2 cm thick) is visible on the top, followed by a green layer of similar thickness. Some of these microbial mats are close to Andean flamingo mound nests which are, in most of the cases, no longer used by the wading birds. The dissection of these mound nests revealed endolithic microbial communities (S2) forming colored strata, similar to the ones present in the evaporite deposits from Laguna Barros Negros (Figure 2). As far as we know, this is the first work to report a microbial ecosystem of this kind in the Central Andes.

Laguna La Punta (∼0.16 km^2^ area) and Laguna Brava (∼0.20 km^2^ area) are two connected lakes from the south of Salar de Atacama that have already been explored (Figure 1). Nevertheless, in both lakes, we have identified previously unknown microbial mats and microbialites. In Laguna La Punta, we have discovered microbial mats associated with vegetation (P1 and P2) that usually display a sequence of (from top to bottom) pink/orange, green and purple colored layers, with cerebroid, snake and globular morphologies, as well as microbialites covered by black biofilms (P3) (Figure 2). Whereas in Laguna Brava, we have discovered red-green-colored microbial mats (B1), oncoids (microbialites that display a concentrically laminated mesostructure) (Logan et al., 1964) covered by black biofilms (B2) and microbialites covered by microbial mats (B3 and B4) that usually display a sequence of (from top to bottom) pink/orange, green and purple colored layers (Figure 2).

The last system prospected was Tilopozo (∼0.34 km^2^ area), a wetland complex located in the south region of Salar de Atacama (Figure 1). Compared to the other systems studied, we have not identified in this environment carbonate or evaporite deposits associated with microbial communities. Instead, we have discovered green floating biofilms (T) covering a great proportion of the wetland surface (Figure 2).

### Mineralogical Analyses

**FIGURE 3:**
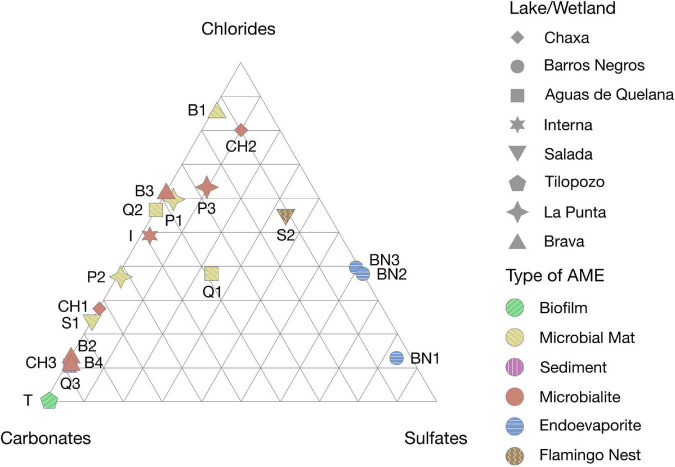
Ternary diagram representing the mineral composition of the AMEs. The shapes and colors indicate the location and type of AME, respectively.

X-ray diffraction analyses were carried out on the samples collected (Supplementary Figures 1–3) to determine and compare the mineralogical composition of the AMEs studied (Figure 3 and Supplementary Table 1). The endoevaporites from Laguna Barros Negros (BN1, BN2 and BN3), as well as the microbial community within the Andean flamingo mound nest from Laguna Salada (S2) are mainly formed by gypsum (ca. 40–65%) and halite (ca. 10–65%), which probably explain their morphological similarities. As the endoevaporite BN3 was located in a drier area compared to the rest of the endoevaporites from Laguna Barros Negros, it also contains a considerable amount (ca. 25%) of anhydrite (CaSO_4_).

The microbial mats from Aguas de Quelana (Q2) and Laguna Salada (S1) are comprised predominantly of halite (ca. 25–65%), aragonite (CaCO_3_) (ca. 25–40%) and calcite (CaCO_3_) (ca. 25–40%). Whereas the microbial mats from Laguna La Punta (P1 and P2) and Laguna Brava (B1), as well as the pink/orange sediment from Aguas de Quelana (Q3), are composed mainly of halite (ca. 10–80%) and aragonite (ca. 10–80%), but do not contain great amounts of calcite (ca. 1–3%). This is also observed in the microbial mat Q1 from Aguas de Quelana, which is deficient in calcite (ca. 1%) but contains a considerable amount of aragonite (ca. 40%), halite (ca. 40%) and gypsum (ca. 25%).

The microbialites from Laguna Chaxa (CH1, CH2 and CH3), Laguna Brava (B3 and B4) and Laguna La Punta (P3), the stromatolite from Laguna Interna (I), and the oncoid from Laguna Brava (B2) are mostly formed by aragonite (ca. 10–80%) and halite (ca. 10–80%). In addition to these minerals, the stromatolite from Laguna Interna (I) contains a great proportion of calcite (ca. 25%), and the microbialite from Laguna La Punta (P3) contains considerable amounts of gypsum (ca. 10%) and opal-A (SiO_2_⋅*n*H_2_O) (ca. 10%).

The floating biofilm from Tilopozo (T) has a mineralogical composition quite different from the rest of the microbial ecosystems studied. Although its principal component is calcite (ca. 65%), it also contains clay (ca. 25%), dolomite (CaMg(CO_3_)_2_) (ca. 25%), quartz (SiO_2_) (ca. 10%), siderite (FeCO_3_) (ca. 10%) and dawsonite (NaAlCO_3_(OH)_2_) (ca. 10%) in great proportion.

### Scanning Electron Microscopy Analyses

**FIGURE 4:**
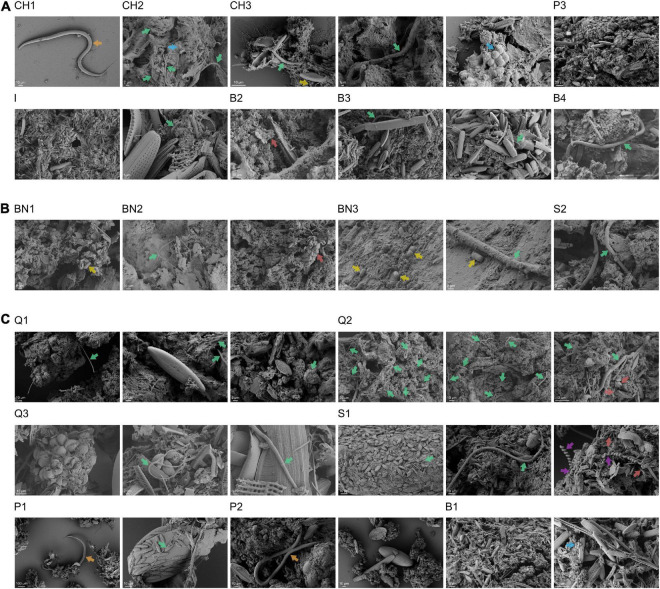
Diatoms, filamentous cyanobacteria and other prokaryotic cells form nano-globular carbonate aggregates in most of the AMEs. **(A)** SEM images of the microbialites. CH1, CH2 and CH3, Microbialites from Laguna Chaxa; P3, Microbialite from Laguna La Punta; I, Microbialite from Laguna Interna; B2, B3 and B4, Microbialites from Laguna Brava. **(B)** SEM images of the endolithic microbial communities. BN1, BN2 and BN3, Endoevaporites from Laguna Barros Negros; S2, Microbial community within an Andean flamingo mound nest from Laguna Salada. **(C)** SEM images of the microbial mats and the pink/orange sediment. Q1 and Q2, Microbial mats from Aguas de Quelana; Q3, Sediment-associated microbial community from Aguas de Quelana; S1, Microbial mat from Laguna Salada; P1 and P2, Microbial mats from Laguna La Punta; B1, Microbial mat from Laguna Brava. Different recognized structures are marked with an arrow. Green arrow, Filamentous cyanobacterium; Blue arrow, Coccus; Yellow arrow, Bacillus; Purple arrow, Spirochete; Orange arrow, Nematode; Red arrow, Mineral grain.

Scanning electron microscopy analyses were performed on the samples to study the association of the prokaryotic and eukaryotic microorganisms with the minerals present in the AMEs studied (Figure 4 and Supplementary Figures 4–6). The SEM images obtained from the microbialite samples (Figure 4A and Supplementary Figure 4) revealed the presence of diatoms (*Bacillariophyceae*), filamentous cyanobacteria and other prokaryotic cells (cocci and bacilli) forming nano-globular carbonate aggregates, which suggests their participation in the mineral precipitation process (Gomez et al., 2018). Based on morphological characteristics, we could identify at least four families of diatoms: *Achnanthaceae* (*Achnanthes* spp.), *Bacillariaceae* (*Nitzschia* spp.), *Catenulaceae* (*Halamphora* spp.), and *Naviculaceae* (*Navicula* spp.). A multicellular eukaryotic organism was also identified in one of the microbialite samples (CH1). This was a free-living roundworm (*Nematoda*) that, based on the shape of its mouth (hollow tube) (Supplementary Figure 4), feeds on the prokaryotes that cover the microbialites (McSorley, 1997).

The SEM images of the endoevaporites and the endolithic microbial community inhabiting the Andean flamingo mound nest (Figure 4B and Supplementary Figure 5) also revealed the presence of diatoms, filamentous cyanobacteria and other prokaryotic cells (bacilli). In this case, the identified families of diatoms were *Achnanthaceae* (*Achnanthes* spp.) and *Naviculaceae* (*Navicula* spp.). But compared to the microbialite systems, the number of diatoms and filamentous cyanobacteria in these systems seemed to be lower.

In the microbial mats and pink/orange sediment (Figure 4C and Supplementary Figure 6), the SEM images showed a great number of diatoms, filamentous cyanobacteria, and other prokaryotic cells (cocci and spirochetes). Like in the microbialites, these microorganisms form aggregates with precipitation of nano-globular carbonates, suggesting their participation in the mineral precipitation process (Gomez et al., 2018). The families of diatoms identified in the microbial mats were *Achnanthaceae* (*Achnanthes* spp.), *Bacillariaceae* (*Nitzschia* spp.), *Catenulaceae* (*Halamphora* spp.), *Mastogloiaceae* (*Mastogloia* spp.), *Naviculaceae* (*Navicula* spp.), and *Surirellaceae* (*Surirella* spp.), while the families of diatoms identified in the pink/orange sediment were *Achnanthaceae* (*Achnanthes* spp.), *Bacillariaceae* (*Nitzschia* spp.), *Catenulaceae* (*Halamphora* spp.), *Mastogloiaceae* (*Mastogloia* spp.), *Naviculaceae* (*Navicula* spp.), *Sellaphoraceae* (*Fallacia* spp.), and *Surirellaceae* (*Surirella* spp.). Free-living roundworms were also observed in the microbial mats (P1 and P2), supporting their role as bacterivorous in these systems.

### Taxonomy-Based Analyses

**FIGURE 5:**
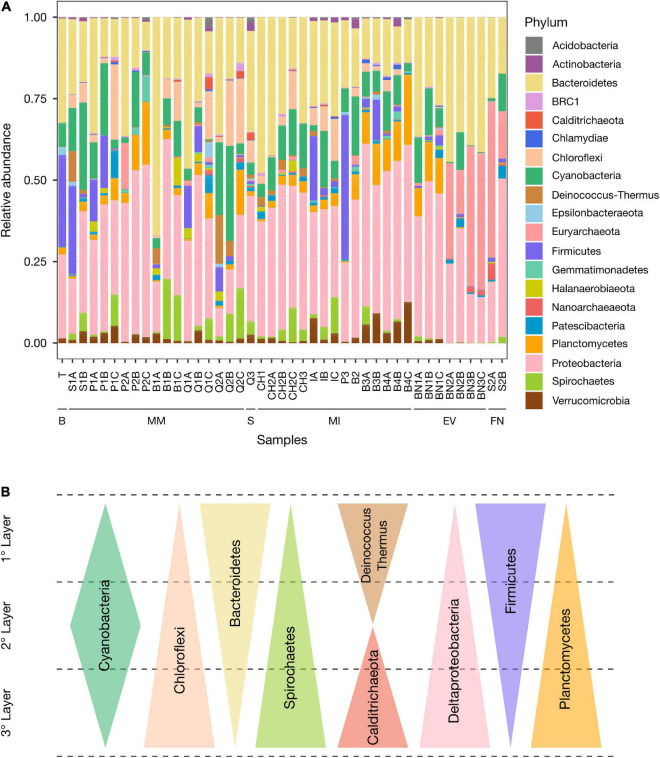
Taxonomic composition of prokaryotes in the AMEs. **(A)** Relative abundance of the twenty major phyla in the different samples. B, Biofilm; MM, Microbial mat; S, Sediment; MI, Microbialite; EV, Endoevaporite; FN, Andean flamingo mound nest. **(B)** Graphical summary of taxa depth distributions observed in the microbial mats.

Molecular diversity analyses based on the small subunit ribosomal (SSU) rRNA of *Bacteria* and *Archaea* were carried out on the samples (Table 1) to determine the prokaryotic composition of the AMEs studied. The results revealed that the taxonomic composition of prokaryotes not only differs between types of AMEs, but also between their layers (Figure 5).

The dominant phyla in the majority of the microbial mats are *Bacteroidetes* (ca. 8.78–66.95%) and *Proteobacteria* (ca. 8.59–52.96%), followed by *Cyanobacteria* (ca. 0.41–29.15%), *Firmicutes* (ca. 0.02–27.19%), *Chloroflexi* (ca. 0–23.10%), and *Spirochetes* (ca. 0.02–18.44%). *Planctomycetes* (ca. 0.48–19.27%) and *Verrucomicrobia* (ca. 0.41–5.33%) phyla are also well-represented (Figure 5A). Within the phylum *Proteobacteria*, the most abundant classes are *Alphaproteobacteria* (ca. 5.23–31.23%), *Gammaproteobacteria* (ca. 1.09–27.24%), and *Deltaproteobacteria* (ca. 0.35–17.29%) (Supplementary Figures 7, 8). While in the phylum *Bacteroidetes*, the most abundant classes are *Bacteroidia* (ca. 0.54–62.70%) and *Rhodothermia* (ca. 0.38–29.85%) (Supplementary Figures 7, 9).

In most carbonate microbialites, *Proteobacteria* (ca. 24.28–50.00%) and *Bacteroidetes* (ca. 11.14–47.73%) are the dominant phyla, followed by *Cyanobacteria* (ca. 1.87–18.08%) and *Chloroflexi* (ca. 0.01–12.89%). *Planctomycetes* (ca. 0.51–21.55%) and *Verrucomicrobia* (ca. 0.30–12.59%) phyla are also abundant, especially in Laguna Brava samples. Contrary to microbial mats, the phylum *Firmicutes* is underrepresented in the majority of the microbialites, except in the ones from Laguna La Punta (P3), Laguna Interna (I) and Laguna Brava (B3) (ca. 44.37%, 1.84–19.83%, and 2.68–12.45%, respectively) (Figure 5A). Within the phylum *Proteobacteria*, the most abundant classes are *Alphaproteobacteria* (ca. 10.97–32.96%), *Gammaproteobacteria* (ca. 3.74–24.20%) and *Deltaproteobacteria* (ca. 0.62–18.12%) (Supplementary Figures 7, 8). Whereas in the phylum *Bacteroidetes*, the most abundant classes are *Bacteroidia* (ca. 1.82–29.32%) and *Rhodothermia* (ca. 0.59–26.29%) (Supplementary Figures 7, 9). Like in microbial mats and microbialites, the dominant phyla in the pink/orange sediment are *Proteobacteria* (ca. 38.47%) and *Bacteroidetes* (ca. 31.01%), followed by *Chloroflexi* (ca. 6.79%), *Spirochetes* (ca. 4.15%), and *Cyanobacteria* (ca. 3.74%) (Figure 5A).

The taxonomic composition of gypsum endoevaporites differs greatly from that of microbial mats and microbialites. *Proteobacteria* (ca. 14.03–48.24%), *Bacteroidetes* (ca. 21.29–44.39%), and *Euryarchaeota* (ca. 0.40–43.04%) phyla dominate the majority of these microbial ecosystems. The *Cyanobacteria* phylum is also abundant (ca. 0.24–14.36%), but in comparison to microbial mats and microbialites, it is less represented (Figure 5A). Within the phylum *Proteobacteria*, the most abundant classes are *Alphaproteobacteria* (ca. 8.39–23.79%) and *Gammaproteobacteria* (ca. 4.58–23.04%). *Deltaproteobacteria* class is present, but underrepresented (ca. 1.06–2.63%) (Supplementary Figures 7, 8). The most abundant classes in the *Bacteroidetes* phylum are *Rhodothermia* (ca. 20.40–41.99%) and *Bacteroidia* (ca. 0.88–4.98%). Interestingly, in all the endoevaporites, *Rhodothermia* class is more abundant than *Bacteroidia* class. However, in most of the microbial mats and microbialites, *Bacteroidia* class is more abundant than *Rhodothermia* class (Supplementary Figures 7, 9). Within the *Euryarchaeota* phylum, the dominant class is *Halobacteria* (ca. 0.40–43.05%) (Supplementary Figure 7). Another interesting observation is the low proportion of *Firmicutes* (ca. 0.06–3.05%), *Spirochetes* (ca. 0.02–1.39%), *Verrucomicrobia* (ca. 0.02–1.23%), *Chloroflexi* (ca. 0.00–0.39%), and *Deinococcus-Thermus* (0.01–0.29%) phyla, which are normally well-represented in the microbial mats and microbialites. The taxonomic composition of the Andean flamingo mound nest resulted to be similar to that of gypsum endoevaporites, with *Proteobacteria* (ca. 18.45–48–63%), *Euryarchaeota* (ca. 14.56–47.94%) and *Bacteroidetes* (ca. 17.27–24.60%) as the dominant phyla (Figure 5A).

Regarding the taxonomic depth distributions (Figure 5B), in the microbial mats, *Deinococcus-Thermus* (1° layer: 0–14.93%, 2° layer: 0–4.66%, 3° layer: 0–0.51%), *Firmicutes* (1° layer: 0.09–27.19%, 2° layer: 0.02–16.16%, 3° layer: 0.03–0.90%) and *Bacteroidetes* (1° layer: 23.04–66.95%, 2° layer: 11.79–25.72%, 3° layer: 8.78–18.78%) taxa normally reduce their abundance with depth. While *Calditrichaeota* (1° layer: 0–0.22%, 2° layer: 0–0.12%, 3° layer: 0–3.76%), *Chloroflexi* (1° layer: 0–3.98%, 2° layer: 0–20.11%, 3° layer: 0.05–23.10%), *Spirochetes* (1° layer: 0.02–1.98%, 2° layer: 0.08–18.44%, 3° layer: 0.02–15.43%), *Deltaproteobacteria* (1° layer: 0.35–2.73%, 2° layer: 1.48–5.82%, 3° layer: 5.68–17.29%), and *Planctomycetes* (1° layer: 0.48–3.09%, 2° layer: 1.63–10.83%, 3° layer: 2.17–19.27%) taxa increase it. The *Cyanobacteria* phylum, contrary to other taxa, is more abundant in the middle layers than in the upper or lower ones (1° layer: 1.73–22.32%, 2° layer: 4.55–29.15%, 3° layer: 0.41–10.90%). With regard to *Gammaproteobacteria* (1° layer: 1.09–26.45%, 2° layer: 2.18–27.03%, 3° layer: 1.75–27.24%) and *Alphaproteobacteria* (1° layer: 5.79–22.04%, 2° layer: 5.24–31.22%, 3° layer: 5.79–19.38%) taxa, no common patterns are observed. Although the microbialites CH2, I, B3, and B4 are covered by microbial mats, their taxa do not present depth-related distribution patterns. In the endoevaporites and the Andean flamingo mound nest, the only depth-related distribution pattern observed is the abundance increase of *Patescibacteria* phylum with depth (1° layer: 0–1.15%, 2° layer: 0.29–3.90%, 3° layer: 0.51–2.58%).

### Diversity Analyses

**FIGURE 6:**
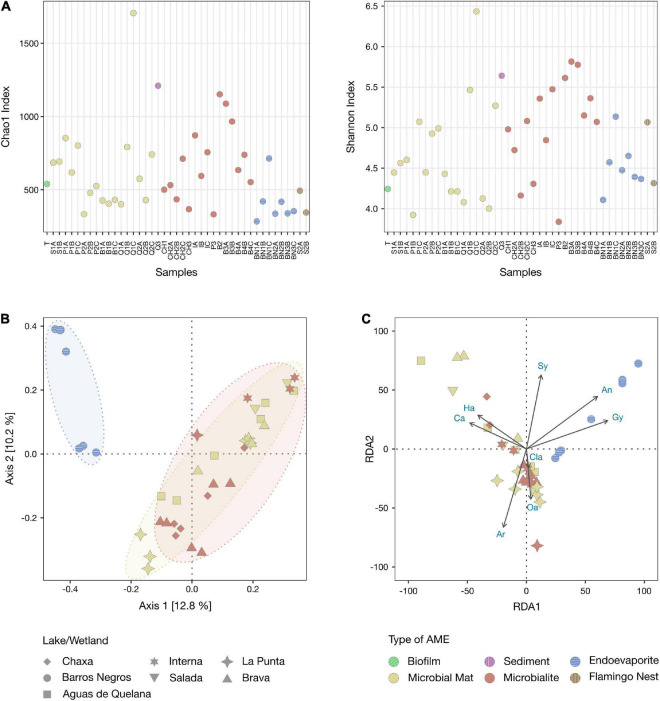
Diversity analyses of the AMEs. **(A)** Comparison of alpha diversity metrics between samples. Left, richness estimates (Chao1 index) for the different samples. Right, diversity estimates (Shannon index) for the different samples. **(B)** Principal coordinate analysis (PCoA) based on Bray-Curtis dissimilarity between endoevaporite, microbial mat, and microbialite samples. **(C)** Redundancy analysis (RDA) of the relationship between minerals and endoevaporite, microbial mat, and microbialite samples. Arrows indicate the direction and magnitude of minerals associated with the samples. Ha, Halite; Ca, Calcite; Ar, Aragonite; Oa, Opal-A; Cla, Clay; Gy, Gypsum; An, Anhydrite; Sy, Sylvite.

Alpha diversity metrics (Chao1 and Shannon indexes) were estimated, after even subsampling (rarefaction), to determine the community structure of each sample (Figure 6A and Supplementary Table 2). The comparison of these metrics, evaluated with Wilcoxon rank-sum test (Mann-Whitney) (Wilcoxon, 1945), revealed that both richness and diversity do not differ significantly (*P* > 0.05) between types of AMEs, nor between layers.

Beta diversity was assessed through a principal coordinate analysis (PCoA) based on Bray-Curtis dissimilarity between endoevaporite, microbial mat, and microbialite samples (Figure 6B). Floating biofilm, sediment and Andean flamingo mound nest samples were not included as random factors as they do not present replicates. The PCoA plot showed a clear separation of the samples based on the type of AME (PERMANOVA, *R*^2^ = 0.152, *P* < 0.001) and the site (PERMANOVA, *R*^2^ = 0.335, *P* < 0.001) (Supplementary Table 3), with the largest variation observed between gypsum endoevaporites, and carbonate mats and microbialites (Axis 1, Figure 6B). Therefore, a redundancy analysis (RDA) was carried out to evaluate further the relationship between the mineralogy and the community composition of the samples (Figure 6C). The RDA plot confirmed what was observed in the PCoA analysis. Gypsum (PERMANOVA, *P* < 0.001) and anhydrite (PERMANOVA, *P* < 0.01) compositions are significantly correlated with the community composition of the endoevaporites, while calcite (PERMANOVA, *P* < 0.001) and aragonite (PERMANOVA, *P* < 0.001) compositions are significantly correlated with the community composition of the mats and microbialites (Supplementary Table 4).

## Discussion

The exploration of high-altitude lakes and wetlands in the middle-east and south-east regions of Salar de Atacama revealed a great number of previously undescribed AMEs, including floating biofilms, sediment-associated microbial communities, microbial mats, microbialites, endoevaporites and endolithic microbial communities inhabiting Andean flamingo mound nests. Nevertheless, not all types of AMEs were found in every prospected environment. Floating biofilms were described in Tilopozo; sediment-associated microbial communities were reported in Laguna Salada; microbial mats were found in Aguas de Quelana, Laguna Salada, Laguna La Punta and Laguna Brava; microbialites were observed in Laguna Chaxa, Laguna Interna, Laguna La Punta and Laguna Brava; endoevaporites were detected in Laguna Barros Negros; and endolithic microbial communities inhabiting Andean flamingo mound nests were discovered in Laguna Salada. The presence of different types of AMEs in the lakes and wetlands studied could be partially explained by their distinct environmental and geochemical conditions (Stivaletta et al., 2011; Gomez et al., 2014; Suosaari et al., 2016; Bougeault et al., 2020; Vignale et al., 2021). Among these AMEs, the endolithic microbial community inhabiting the Andean flamingo mound nest could be considered one of the most interesting findings. Thus far, endoevaporites were the only microbial endolithic communities reported in the Central Andes environments (Vignale et al., 2021). Therefore, this discovery opens the possibility of finding new types of microbial endolithic communities in the salt flats of the region. Endolithic microorganisms are often the main form of life in the most extreme terrestrial climates, and thus could provide important clues about ancient life on the Earth or elsewhere in the Solar System (Walker et al., 2005).

The XRD analyses revealed diverse mineralogical compositions in the AMEs studied. Microbial mats and microbialites found in Laguna Chaxa, Aguas de Quelana, Laguna Interna, Laguna Salada, Laguna La Punta and Laguna Brava are mainly formed by halite and calcium carbonate, like the majority of the mats and microbialites previously described in Salar de Atacama (Thiel et al., 2010; Farías et al., 2014; Farias et al., 2017; Farías, 2020). However, in all the microbialites, calcium carbonate is mostly present as aragonite. While, in some microbial mats, calcium carbonate is present both as aragonite and calcite. There are several factors that could favor the precipitation of calcium carbonate as calcite or as aragonite. The presence of sulfate (SO_4_^2–^) or magnesium (Mg^2+^) in solution (Berner, 1975; Walter, 1986), as well as a high rate of carbonate precipitation (Given and Wilkinson, 1985), could favor precipitation of aragonite. Whereas, the presence of phosphate (PO_4_^3–^) or iron (Fe^2+^) in solution (Meyer, 1984; Walter, 1986) could favor precipitation of calcite. As we do not have measurements of these parameters, we cannot be certain which factor favors the precipitation of calcium carbonate as calcite or as aragonite in the microbial mats and microbialites from this study. Therefore, water geochemical analyses should be carried out in the future to assess this interrogation. Contrary to microbial mats and microbialites, the endoevaporites from Laguna Barros Negros are mainly formed by gypsum and halite, like the previously studied endoevaporites from Laguna Tebenquiche (Farías et al., 2014; Fernandez et al., 2016). This mineral composition is also observed in the Andean flamingo mound nest from Laguna Salada, which could be explained by the fact that Andean flamingos build their mound nests with mud from the salt flat (Brown and King, 2005).

The SEM images of the microbial mats and microbialites studied revealed filamentous cyanobacteria and other prokaryotic cells (cocci, bacilli, and spirochetes) associated with diatom frustules forming nano-globular carbonate aggregates. Diatoms and cyanobacteria fix carbon dioxide and release oxygen through photosynthesis, which increases alkalinity and promotes carbonate precipitation (Dupraz and Visscher, 2005; Dupraz et al., 2009). The large amounts of EPS produced by them also influence carbonate precipitation, as the EPS matrix provides a template for carbonate nucleation (Dupraz and Visscher, 2005; Dupraz et al., 2009). Therefore, the formation of these nano-globular carbonate aggregates might suggest their participation in the mineral precipitation process (Gomez et al., 2018). Although diatoms and cyanobacteria seem to be key structural components in all the AMEs studied, a lower number of them were observed in the endoevaporites than in the microbial mats and microbialites. Microorganisms that colonize gypsum evaporites extract water from the gypsum crystals to withstand desiccation (Huang et al., 2020). However, under severe xeric conditions, this mechanism might not be enough for the development of diatoms and cyanobacteria, which could explain their lower abundance in the evaporite deposits. This hypothesis is supported by the lower abundance of cyanobacteria in the endoevaporite BN3 (ca. 0.24–0.28%), which is rich in anhydrite, than in the endoevaporites BN1 and BN2 (ca. 5.72–14.36% and 0.35–11.57%, respectively). The growth of diatoms in the gypsum evaporites could also be affected by a low silicon concentration in these systems (Martin-Jezequel et al., 2000). Most genera of diatoms identified in this work (*Navicula*, *Halamphora*, *Nitzschia*, *Achnanthes*, *Surirella*, and *Mastogloia*) have also been reported in other AMEs from the Central Andes (Farías et al., 2013, 2014; Rasuk et al., 2014; Albarracín et al., 2015; Gomez et al., 2018), which suggests a wide distribution of these taxa in the Central Andes environments.

Regarding the prokaryotic composition, differences have been observed between gypsum endoevaporites and carbonate mats and microbialites. Gypsum endoevaporites are dominated by *Proteobacteria*, *Bacteroidetes*, and *Euryarchaeota* phyla. The high halotolerance of *Bacteroidetes* and *Euryarchaeota* species could explain their great abundance in this type of AME (Farías et al., 2014). On the contrary, *Firmicutes*, *Spirochetes*, *Verrucomicrobia*, *Chloroflexi*, and *Deinococcus*-*Thermus* phyla are underrepresented. Probably, the moisture conditions of the evaporite deposits are not suitable for the correct growth of these microorganisms. Interestingly, a similar taxonomic composition has been observed in other gypsum endoevaporites from Salar de Atacama (Farías et al., 2014; Fernandez et al., 2016), which suggests a correlation between the microbiota and the mineralogy. Carbonate mats and microbialites are dominated by *Bacteroidetes* and *Proteobacteria* phyla, followed by *Cyanobacteria*, *Chloroflexi*, *Planctomycetes*, and *Verrucomicrobia* phyla. Oxygenic and anoxygenic phototrophs from *Cyanobacteria*, *Chloroflexi*, and *Proteobacteria* phyla, as well as sulfate reducers from *Deltaproteobacteria* class, increase alkalinity and promote carbonate precipitation, which could explain their great abundance in these types of AMEs (Dupraz et al., 2009). Other carbonate-rich mats and microbialites from Salar de Atacama (Farías et al., 2014; Fernandez et al., 2016) present a similar taxonomic composition, which further suggests a correlation between the microbiota and the mineralogy. Due to the previous observations, we performed a RDA analysis evaluated with a PERMANOVA test, and confirmed that gypsum, anhydrite, calcite and aragonite compositions are significantly correlated with the community composition of the samples. In the future, this could help us better understand the role of prokaryotes in the formation of the AMEs (Dupraz and Visscher, 2005; Dupraz et al., 2009).

The taxonomy-based analyses also revealed that certain microbial groups are arranged in a vertically organized structure in the microbial mats. This structure is normally determined by steep and fluctuating gradients of light, oxygen, hydrogen sulfide and pH, among other physicochemical gradients (Dupraz and Visscher, 2005; Dupraz et al., 2009). *Bacteroidetes*, *Firmicutes*, and *Deinococcus*-*Thermus* phyla are more abundant in the upper layers, probably due to the high resistance of their species to desiccation and/or ionizing radiation (Makarova et al., 2001; Rainey et al., 2005; Galperin, 2013; Yu et al., 2015). Species of the *Cyanobacteria* phylum are generally found in great proportion in the intermediate layers, a few millimeters beneath the surface, where they are protected from the harsh conditions of the environment to perform oxygenic photosynthesis (Stal, 1995). Whereas species of the *Chloroflexi*, *Deltaproteobacteria*, *Planctomycetes*, *Spirochetes*, and *Calditrichaeota* taxa are more abundant in the lower layers, which are normally anoxic. This could be partially explained by the metabolic activities carried out by them. *Chloroflexi* species consume fermentation products and perform anoxygenic photosynthesis (Lee et al., 2014), *Deltaproteobacteria* and *Planctomycetes* species grow anaerobically reducing sulfate and oxidizing ammonium, respectively (Madigan et al., 2017), while *Spirochetes* and *Calditrichaeota* species degrade organic compounds under anoxic conditions (Marshall et al., 2017; Dong et al., 2018).

## Conclusion

The exploration carried out in the middle-east and south-east regions of Salar de Atacama allowed us to identify twenty previously unknown AMEs. The analyses performed on these AMEs revealed that (1) their mineralogical composition consists mainly of sulfates and/or calcium carbonates, the latter mostly present as aragonite; (2) their prokaryotic composition is correlated with their mineralogy; and (3) most harbor numerous diatom frustules forming nano-globular carbonate aggregates with cyanobacteria and other prokaryotic cells, which suggests the participation of these microorganisms in the mineral precipitation process.

Nevertheless, to have a better comprehension of the AMEs in the Salar de Atacama, further analyses need to be performed to confirm the participation (directly or indirectly) of diatoms and other eukaryotes in the mineral precipitation process; determine the seasonal variations of the environmental factors and their effect on the prokaryotic composition; and elucidate the metabolic potential of their microorganisms in terms of resistance to environmental conditions.

This work has expanded our comprehension of the AMEs inhabiting the high-altitude lakes and wetlands from the Central Andes region and we hope it will provide a stimulus to further study and preserve these unique ecosystems.

## Data Availability Statement

The datasets presented in this study can be found in online repositories. The names of the repository/repositories and accession number(s) can be found below: ENA, PRJEB44218.

## Author Contributions

FV conducted the experiments for the project, analyzed data, and drafted the manuscript. DK and AT assisted with bioinformatic analysis. AL assisted with the interpretation of SEM images. DP assisted with mineralogy analysis. EC, NM-H, FN, and MC performed sampling. MF obtained funding for the original project idea, performed sampling and supervised the findings of this work. All authors assisted in the reviewing and editing of the manuscript.

## Conflict of Interest

The authors declare that the research was conducted in the absence of any commercial or financial relationships that could be construed as a potential conflict of interest.

## Publisher’s Note

All claims expressed in this article are solely those of the authors and do not necessarily represent those of their affiliated organizations, or those of the publisher, the editors and the reviewers. Any product that may be evaluated in this article, or claim that may be made by its manufacturer, is not guaranteed or endorsed by the publisher.
