# Supplementary material for: Geobiology of Andean Microbial Ecosystems Discovered in Salar de Atacama, Chile
Source: Front Microbiol. 2021 Oct 28;12:762076. doi: 10.3389/fmicb.2021.762076 (PMC8581658; doi:10.3389/fmicb.2021.762076)
Supplement: Supplementary file 1 [file Data_Sheet_1.PDF]

frontiers

Supplementary Material

1. Supplementary Tables

Supplementary Table S1. Mineralogical composition of the samples.

Sample	Qu	Po	Pl	Cla	Ca	Ar	Do	Si	Da	Ana	Cli	La	Gy	An	Oct	Oa	He	Ha	Sy	Gi
CH1	3			1	1	65												25		
CH2			3			10							10					80		
CH3				3	3	80							1					10		
BN1						3							65					10		
BN2													65	1				40		
BN3	1					1							40	25				40	3	
Q1	1		3		1	40							25			1		40		
Q2					25	25												65		
Q3					3	80							1					10		
I	1				25	40							3			1		65		
S1	3				40	40												25		
S2				1	10	3							40					65		
T	10	1	1	25	65		25	10	10				1		1					
P1					1	40							3					65		
P2					3	65							1					40		
P3	3				3	25							10			10		65		
B1					3	10							1					80	3	
B2					1	65										1		10		
B3	10				1	40												65	1	
B4					1	80							1					10		

Qu = Quartz; Po = Potassium feldspar; Pl = Plagioclase feldspar; Cla = Clay; Ca = Calcite; Ar = Aragonite; Do = Dolomite; Si = Siderite; Da = Dawsonite; Ana = Analcime; Cli = Clinoptilolite; La = Laumontite; Gy = Gypsum; An = Anhydrite; Oct = Opal-CT; Oa = Opal-A; He = Hematite; Ha = Halite; Sy = Sylvite & Gi = Gibbsite.

Supplementary Table S2. Alpha diversity metrics of the samples.

	Richness		Diversity	
	Chao1	ACE	Shannon	Simpson
CH1	500.86	499.54	4.979	0.982
CH2A	531.52	532.40	4.723	0.977
CH2B	434.62	438.98	4.162	0.945
CH2C	711.75	715.90	5.082	0.977
CH3	367.32	369.91	4.307	0.967
BN1A	284.21	284.52	4.109	0.960
BN1B	420.46	421.45	4.572	0.974
BN1C	713.68	718.22	5.136	0.983
BN2A	336.75	337.10	4.474	0.948
BN2B	418.18	416.40	4.652	0.976
BN3B	338.86	338.86	4.392	0.942
BN3C	353.75	354.00	4.368	0.937
Q1A	401.22	406.50	4.080	0.953
Q1B	792.38	796.16	5.464	0.989
Q1C	1706.48	1711.45	6.434	0.996
Q2A	575.25	581.83	4.126	0.950
Q2B	429.50	427.11	4.003	0.920
Q2C	740.74	741.77	5.270	0.989
Q3	1210.29	1223.04	5.640	0.986
IA	871.18	875.66	5.358	0.984
IB	594.45	597.12	4.846	0.976
IC	756.01	764.63	5.474	0.991
S1A	685.00	684.34	4.447	0.968
S1B	692.90	697.38	4.564	0.925
S2A	493.25	495.32	5.066	0.984
S2B	344.71	345.36	4.316	0.961
T	539.06	538.50	4.244	0.959
P1A	852.56	854.64	4.604	0.969
P1B	618.50	619.83	3.923	0.929
P1C	801.75	807.76	5.073	0.981
P2A	334.00	334.55	4.450	0.965
P2B	478.64	479.36	4.928	0.982
P2C	525.38	528.40	4.990	0.986
P3	332.00	330.82	3.839	0.940
B1A	426.98	432.11	4.430	0.960
B1B	405.44	406.56	4.214	0.935
B1C	431.48	437.56	4.214	0.954
B2	1152.01	1143.54	5.614	0.989
B3A	1088.08	1095.17	5.815	0.993
B3B	966.26	972.58	5.776	0.990

Supplementary Table S2. Alpha diversity metrics of the samples. (Continue)

	Richness		Diversity	
	Chao1	ACE	Shannon	Simpson
B4A	634.42	636.67	5.150	0.986
B4B	738.76	741.81	5.363	0.988
B4C	552.41	557.34	5.071	0.987

Supplementary Table S3. PERMANOVA (Adonis) results based on Bray-Curtis dissimilarity.

	d.f.	*SS*	*MS*	*pseudoF*	$R^{2a,b}$
Main effects					
Site (Lake/Wetland)	6	5.467	0.91117	2.6908	0.33534***
Residuals	32	10.836	0.33862		0.66466
Total	38	16.303			1.00000
Type of AME	2	2.4861	1.2430	3.2388	0.15249***
Residuals	36	13.8168	0.3838		0.84751
Total	38	16.3028			1.00000

Abbreviations: d.f., degrees of freedom; SS, sum of squares; MS, mean sum of squares.

[a]Significance values based on 999 permutations.

[b]Significance levels: * < 0.05, ** < 0.01, *** < 0.001.

Supplementary Table S4. ANOVA-like permutation test for the redundancy analysis (RDA).

	d.f.	*V*	*pseudoF*	*Pr(>F)*
Analysis of all the constraints together				
Model	10	21888742	2.4892	0.001***
Residual	28	24621509		
Analysis of each constraint				
Qu	1	731079	0.8314	0.581
Pl	1	913100	1.0384	0.366
Cla	1	1673600	1.9032	0.089
Ca	1	3681583	4.1868	0.001***
Ar	1	2864982	3.2581	0.001***
Gy	1	3638505	4.1378	0.001***
An	1	2693619	3.0632	0.006**
Oa	1	2674495	3.0415	0.009**
Ha	1	1320412	1.5016	0.079
Sy	1	1697367	1.9303	0.019*
Residual	28	24621509		

Abbreviations: d.f., degrees of freedom; V, variance.

[a]Significance values based on 999 permutations.

[b]Significance levels: * < 0.05, ** < 0.01, *** < 0.001.

2. Supplementary Figures

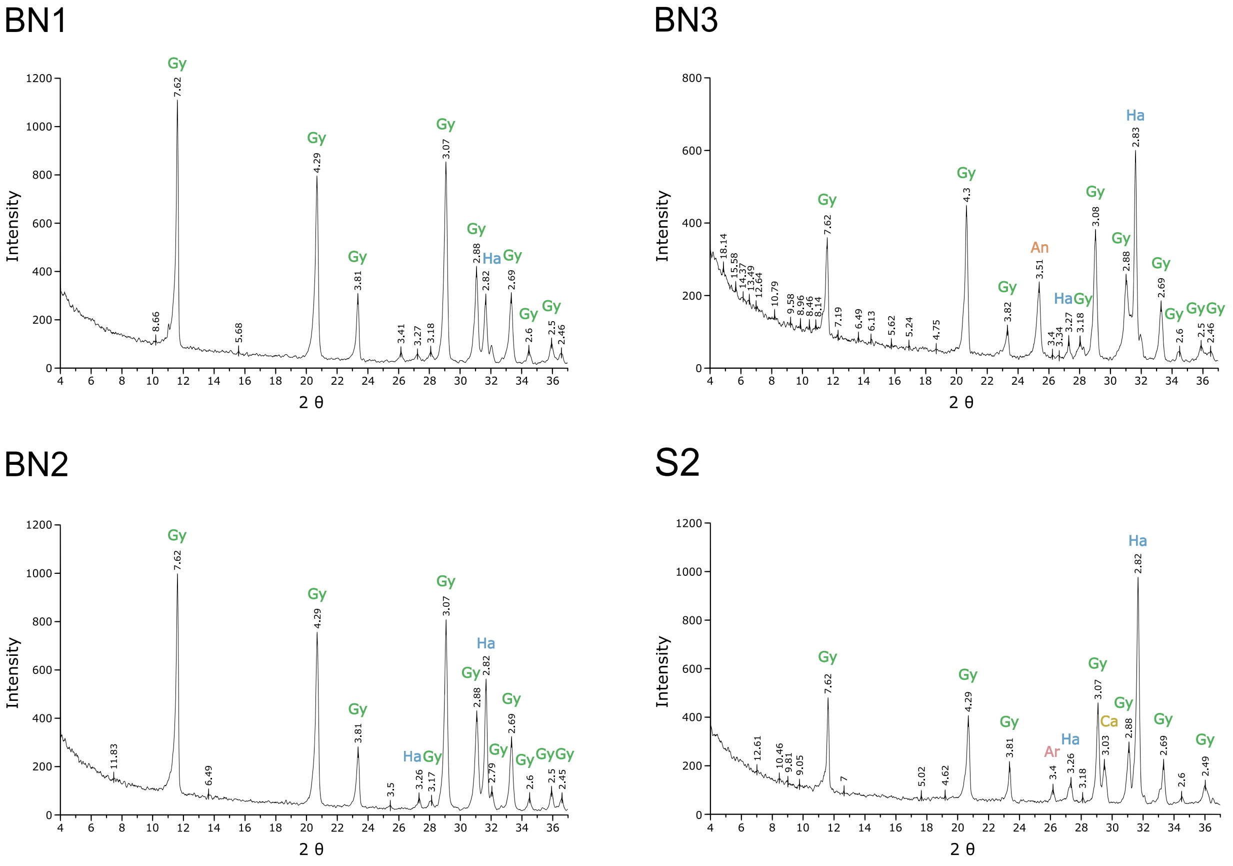

Supplementary Figure S1. XRD patterns of the endoevaporites and the endolithic microbial community inhabiting the Andean flamingo mound nest.

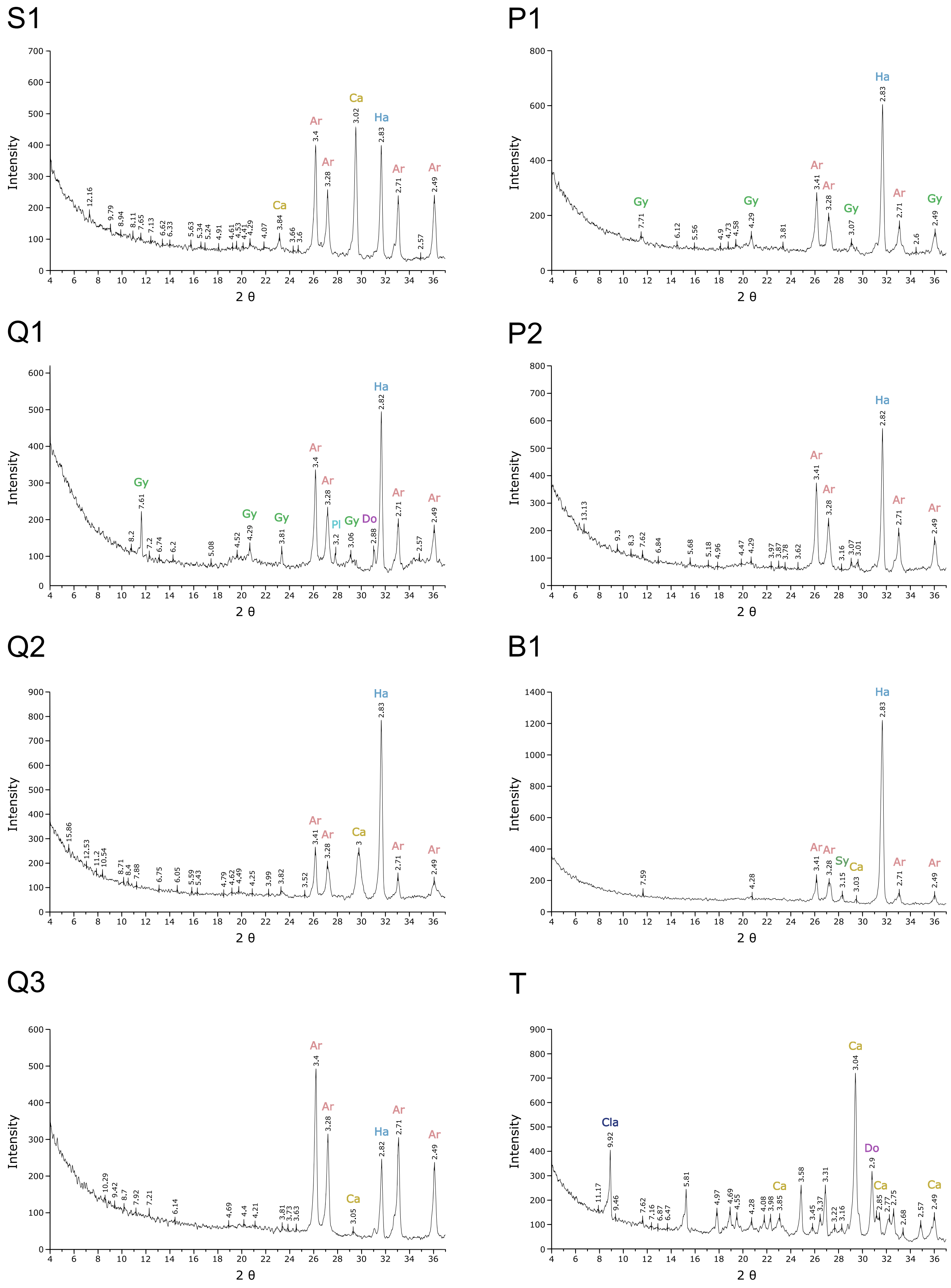

Supplementary Figure S2. XRD patterns of the microbial mats, the pink/orange sediment, and the floating biofilm.

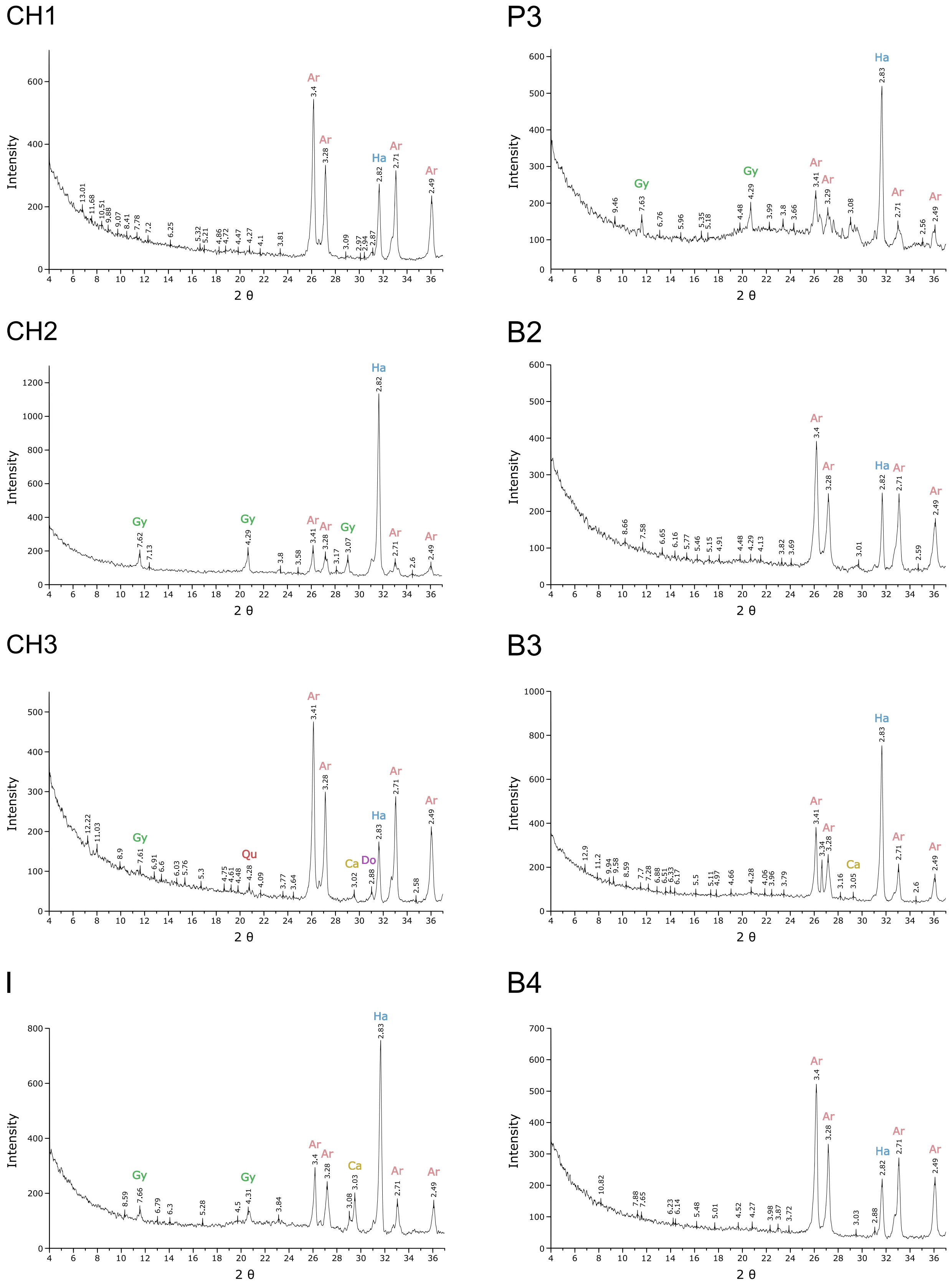

Supplementary Figure S3. XRD patterns of the microbialites.

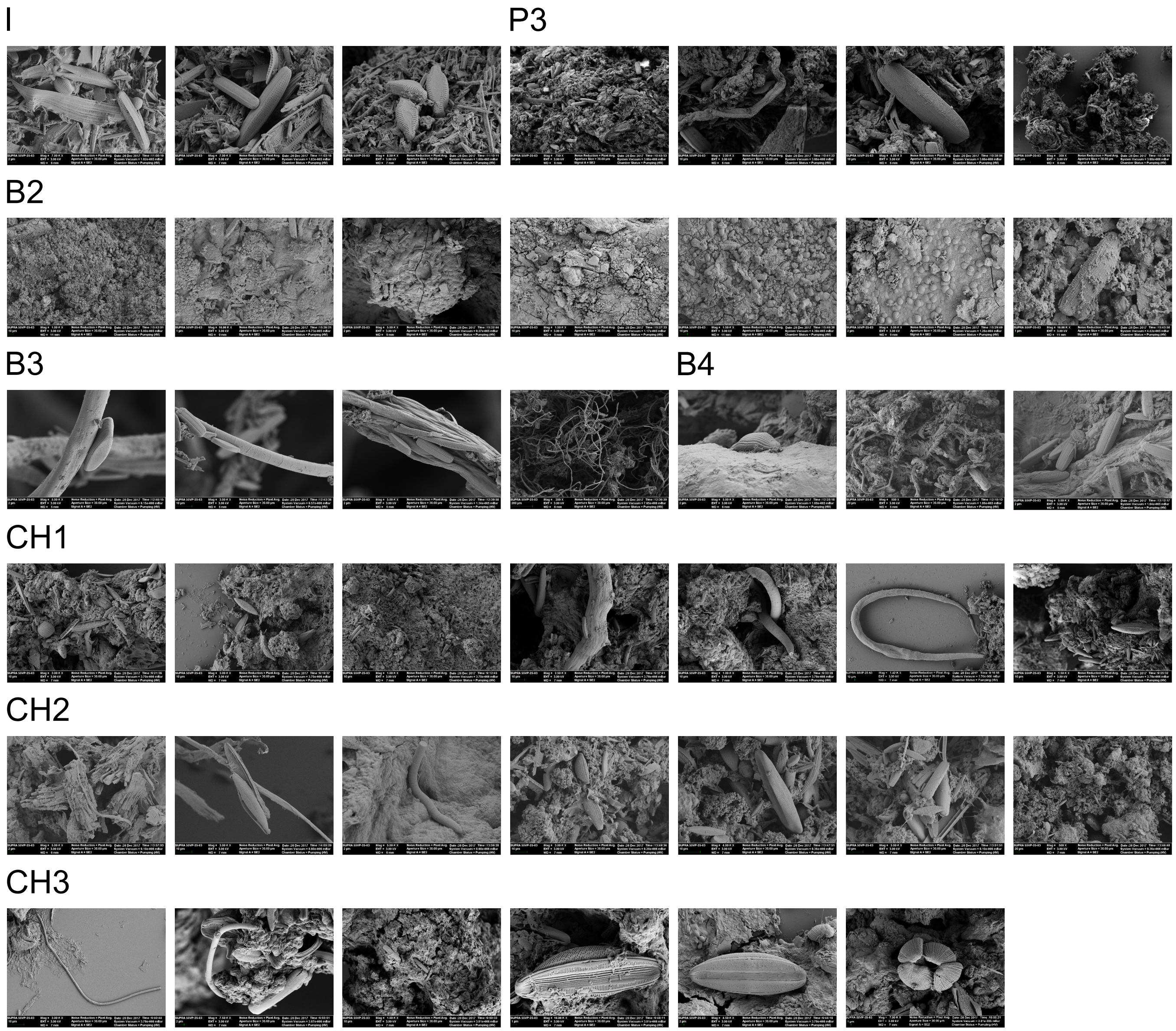

Supplementary Figure S4. Supplementary scanning electron microscopy images of the microbialites.

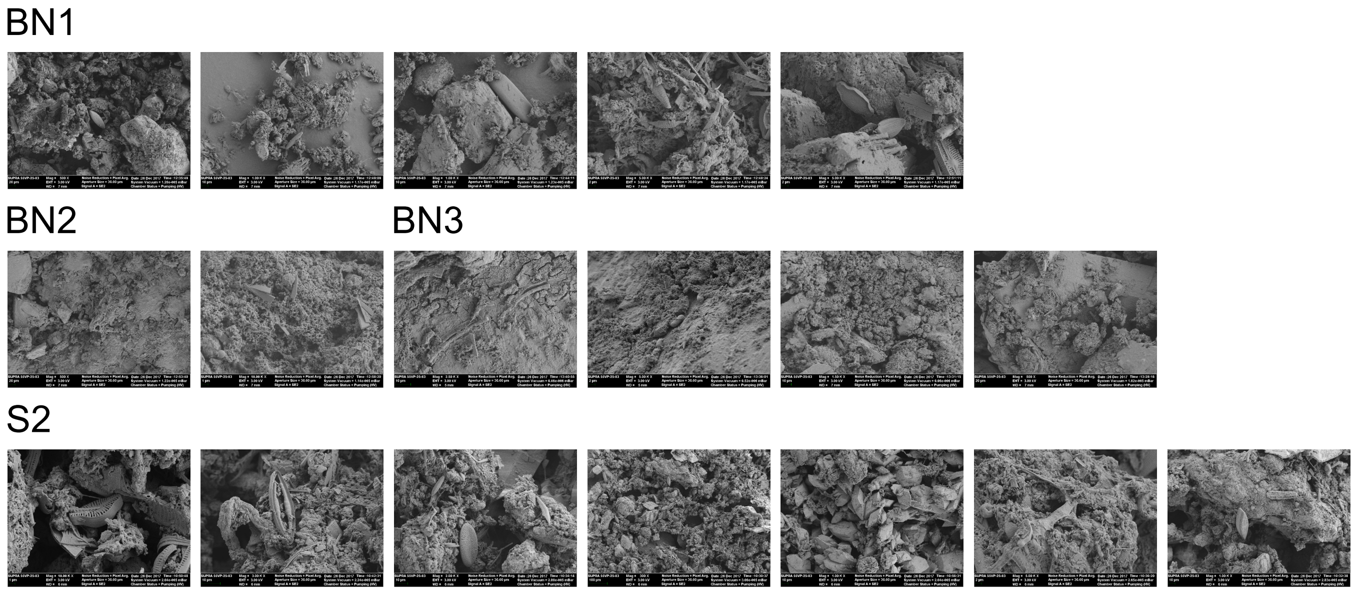

Supplementary Figure S5. Supplementary scanning electron microscopy images of the endoevaporites and the endolithic microbial community inhabiting the Andean flamingo mound nest.

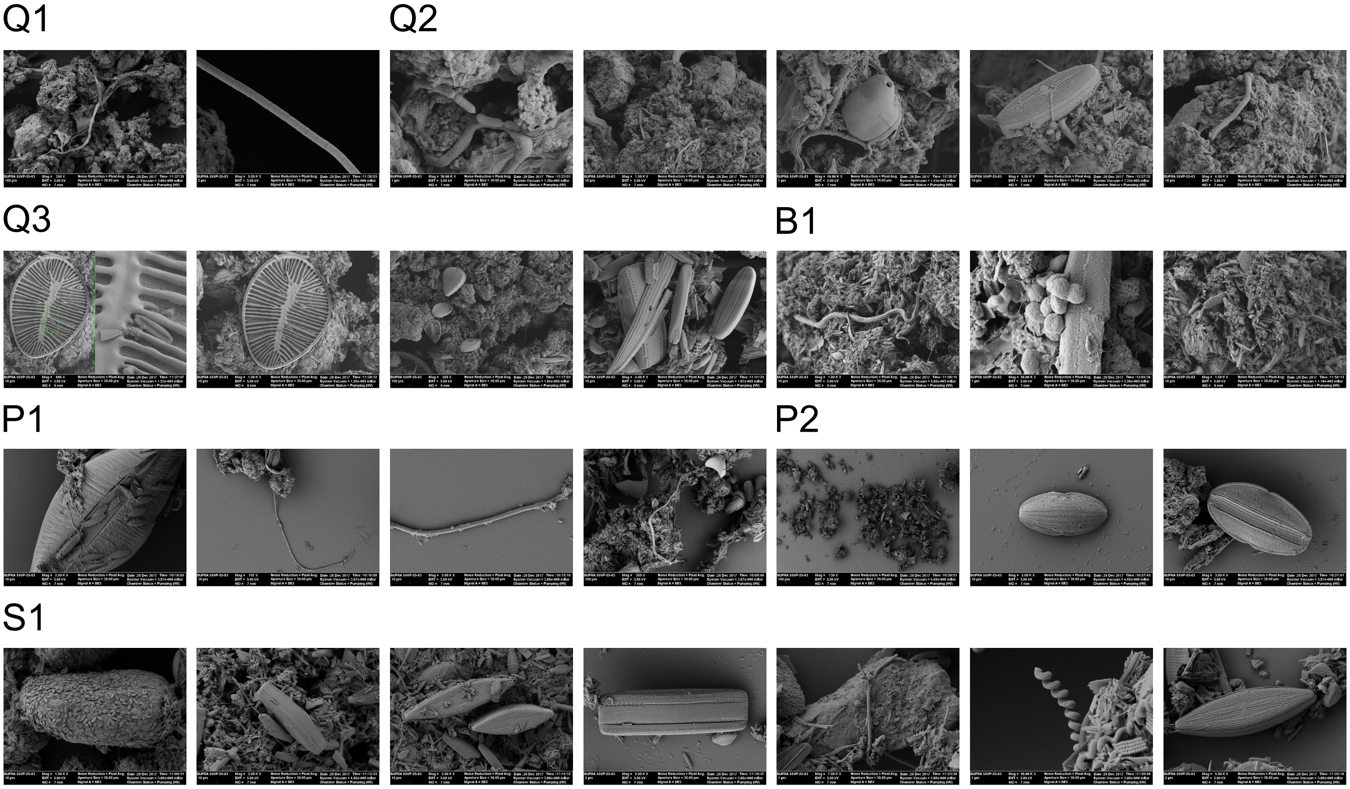

Supplementary Figure S6. Supplementary scanning electron microscopy images of the microbial mats and the pink/orange sediment.

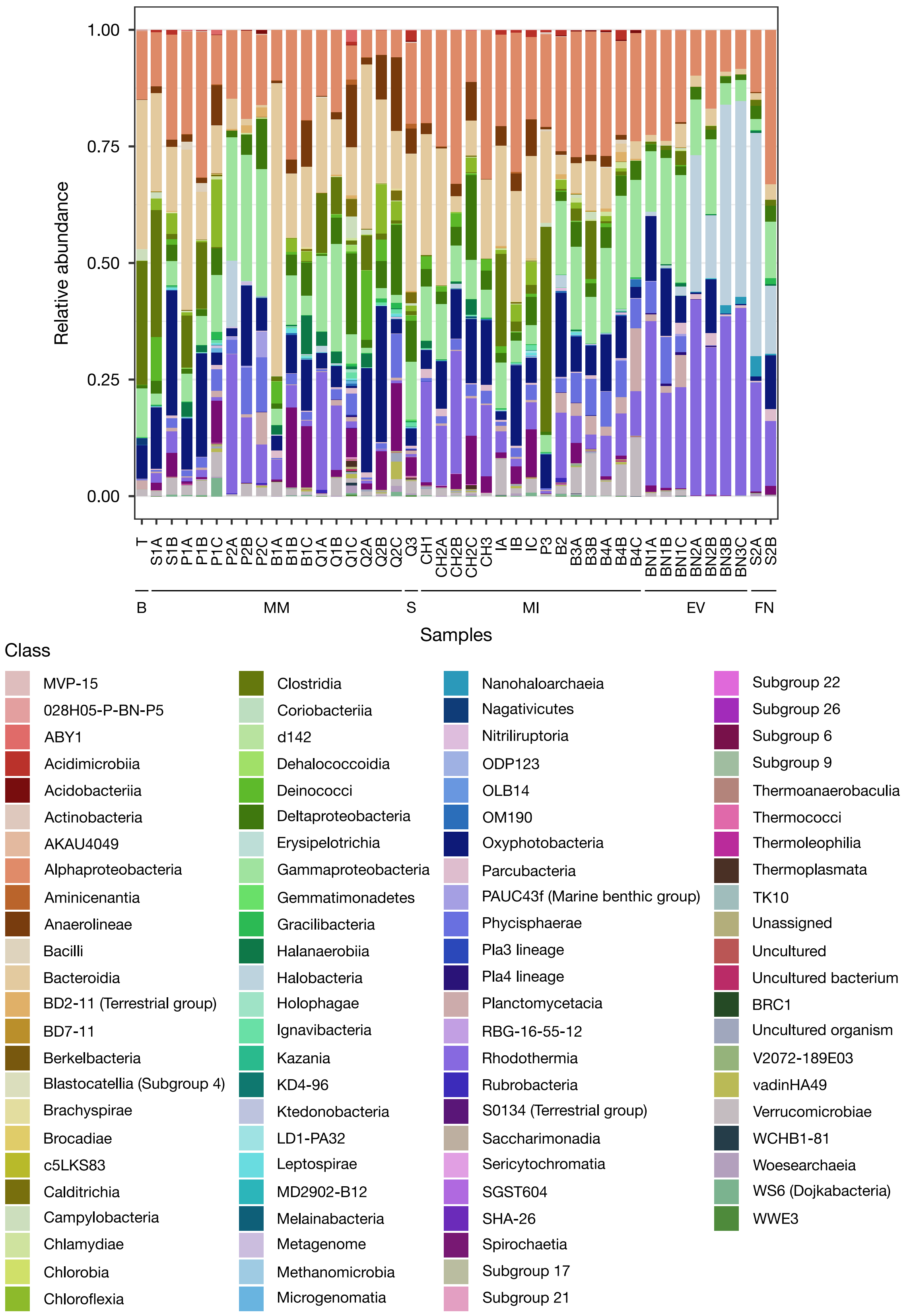

Supplementary Figure S7. Relative abundance of prokaryotic taxa at the class level in the different samples.

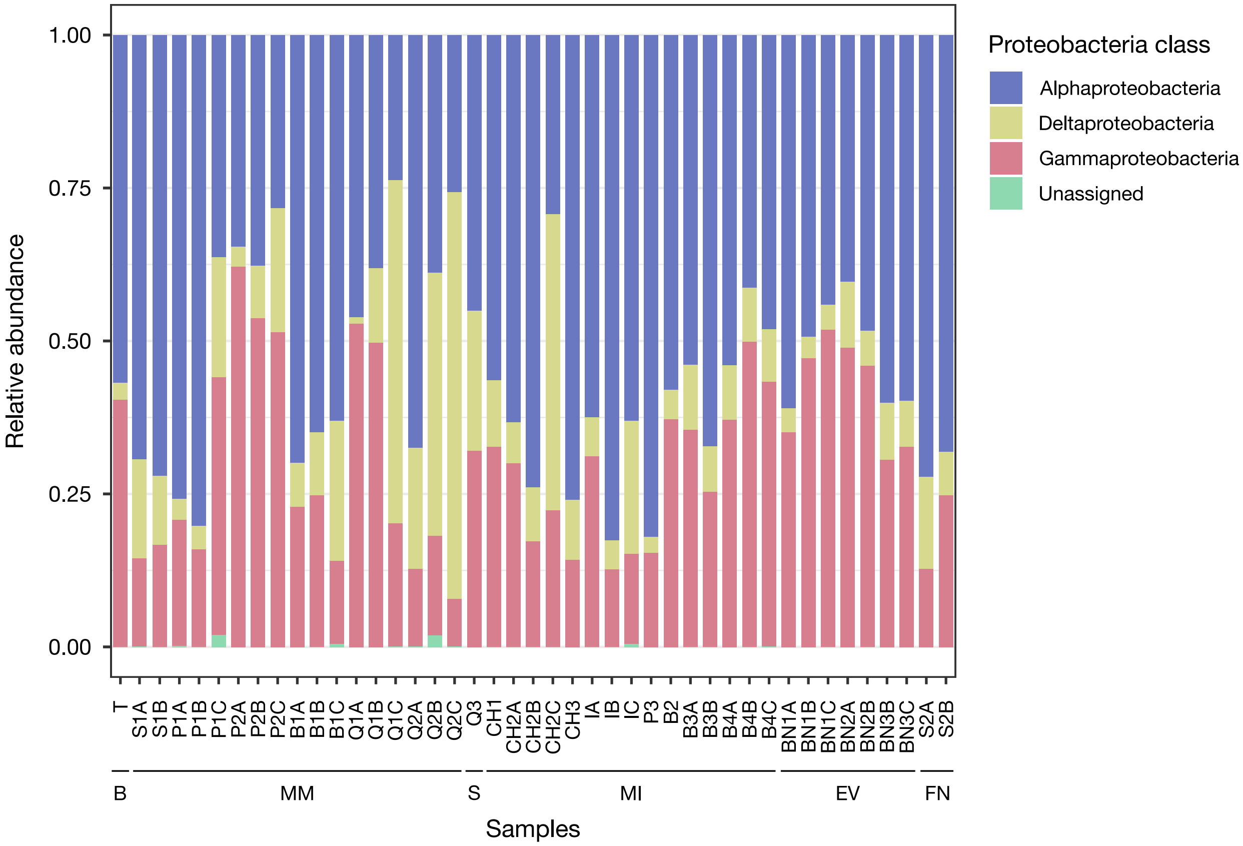

Supplementary Figure S8. Relative abundance of *Proteobacteria* classes in the different samples.

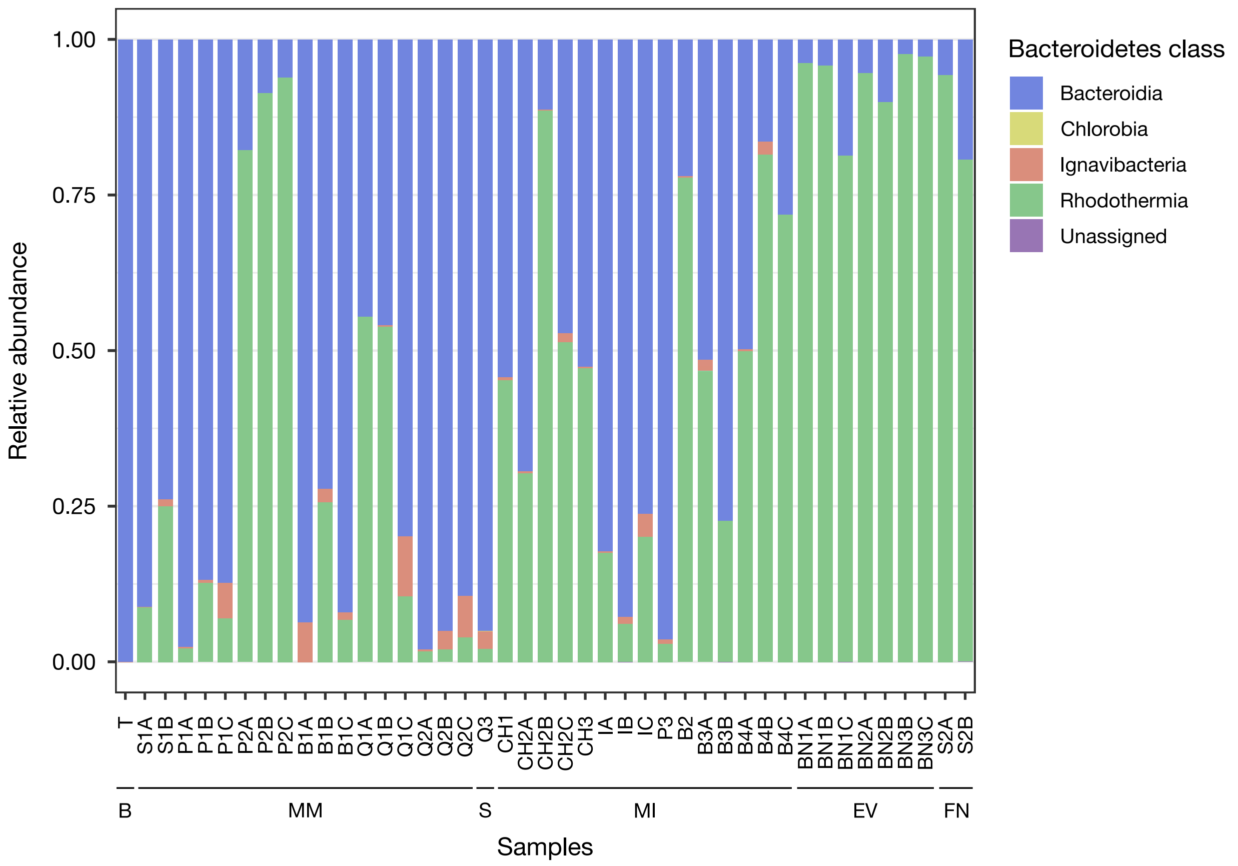

Supplementary Figure S9. Relative abundance of *Bacteroidetes* classes in the different samples.
